# *Babesia* spp. and other pathogens in ticks recovered from domestic dogs in Denmark

**DOI:** 10.1186/s13071-015-0843-0

**Published:** 2015-05-08

**Authors:** Christen Rune Stensvold, Dua Al Marai, Lee O’Brien Andersen, Karen Angeliki Krogfelt, Jørgen Skov Jensen, Kim Søholt Larsen, Henrik Vedel Nielsen

**Affiliations:** Department of Microbiology and Infection Control, Statens Serum Institut, Artillerivej 5, DK—2300 Copenhagen, Denmark; KSL Consulting, Ramløsevej 25, DK—3200 Helsinge, Denmark; Laboratory of Parasitology, Department of Microbiology and Infection Control, Statens Serum Institut, Artillerivej 5, DK—2300 Copenhagen, Denmark

**Keywords:** Zoonosis, Denmark, Parasite, Bacteria, Parasite-vector, Vector-borne, Companion animal, Tick, Fever, Diagnosis

## Abstract

**Background:**

Newly recognized endemic foci for human babesiosis include Europe, where *Ixodes ricinus*, a vector for several species of *Babesia*, is the most commonly identified tick. Vector-based surveillance provides an early warning system for the emergence of human babesiosis, which is likely to be under-reported at emerging sites. In the present study, we set out to screen *I. ricinus* collected from Danish domestic dogs for *Babesia*, in order to identify whether humans in Denmark are exposed to the parasite.

**Findings:**

A total of 661 ticks (*Ixodes* spp.) were collected from 345 Danish domestic dogs during April-September 2011 and pooled, one sample per dog. DNA was extracted from each sample and examined by PCR and sequencing for *Rickettsia* spp., *Borrelia burgdorferi* sensu lato, *Bartonella* spp., *Francisella tularensis*, Candidatus *Neoehrlichia mikurensis*, and *Babesia* spp. In total, 34% of the samples were positive for tick-borne microorganisms potentially pathogenic to humans: *Rickettsia* spp. were detected in 16% of the pools, with 79% being *R. helvetica. Borrelia burgdorferi* sensu lato was found in 15%, with the main species identified as *Borrelia afzelii* (39%). Likewise, 8% of the samples were positive for *Babesia* spp. (*Babesia microti*, 82%; *Babesia venatorum* (‘EU1’), 18%). Lastly, 1% of the samples tested positive for Candidatus *Neoehrlichia mikurensis*, and 0.6% for *Bartonella* spp. No ticks were found to be infected with *Francisella tularensis*.

**Conclusions:**

Our data are in support of endemic occurrence of potentially zoonotic *Babesia* in Denmark and confirms *I. ricinus* as a vector of multiple pathogens of public health concern.

**Electronic supplementary material:**

The online version of this article (doi:10.1186/s13071-015-0843-0) contains supplementary material, which is available to authorized users.

## Findings

Tick-borne pathogens of public health concern include viruses, bacteria, and parasites. The current focus on climate-induced environmental changes potentially favouring the emergence of novel infectious diseases and changes in disease endemicity, boosts the incentive for pathogen surveillance in arthropod vectors and hosts in order to understand the epidemiology and control of these diseases.

One such emerging pathogen is *Babesia*, a parasite first detected in cattle but now a well-known cause of malaria-like illness in humans [1-3]. Newly recognized endemic foci for human babesiosis include Europe [4] where *Ixodes ricinus,* a vector of multiple species of *Babesia,* is common. Apparently the most common tick in Europe [5], *I. ricinus* has a world-wide occurrence and low host specificity, parasitising on a variety of larger mammals, including humans [6].

Human babesiosis should generally be suspected in splenectomised and otherwise immunocompromised individuals with severe febrile illnesses. The first case of human babesiosis in Denmark was reported in 2013 [7] and represented an imported case of *Babesia microti* infection from the United States. There are currently no other reports on autochthonous Danish cases, which could indicate that human exposure to *Babesia* in Denmark is low. Meanwhile, a recent study in the US [8] suggested that vector-based surveillance could provide an early warning system for the emergence of human babesiosis, which is likely to be under-reported at emerging sites. Recently, the presence of *Babesia divergens* and *Babesia venatorum* was demonstrated in Danish *I. ricinus* [9]. Here, we set out to expand on the knowledge of the distribution, prevalence, and species of *Babesia* in ticks collected in Denmark. To gain more information on tick-borne transmitted pathogens in Denmark in general, we moreover sought to identify the prevalence of tick-borne bacteria, including *Borrelia*, Candidatus *Neoehrlichia*, *Bartonella*, *Francisella*, and *Rickettsia*.

The survey was originally developed to identify the prevalence in Denmark of *Dermacentor reticulatus*, a tick commonly found in German dogs [10]. A total of 22 veterinary clinics were instructed by the companies KSL Consulting ApS and Merial Norden A/S to collect ticks from dogs from four regions of Denmark, namely South, Mid-West, and North-West Jutland plus South Funen; these areas were considered to be the most likely places to find *D. reticulatus*, mainly due to the proximity to Germany. Ticks were identified to species level and life cycle stage by morphological analysis [11,12]. For each animal, one or more ticks were collected. In cases where multiple *I. ricinus* ticks were collected per animal, ticks were pooled and analysed as a single sample. Information on sample location (municipality) and date of collection was available.

Bead-beating proved sufficient for DNA extraction when using garnet beads for middle-sized ticks (2–4 mm) and glass beads for small ticks (1–2 mm). Larger ticks had to be cut longitudinally for DNA to be extracted efficiently. However, differential DNA extraction by tick size proved difficult, since several host-tick pools contained ticks of varying sizes. Therefore, longitudinal cutting was selected as the method of choice.

DNA was extracted using QIAamp Mini Kit (QIAGEN, Hilden, Germany) according to the recommendations of the manufacturer. A variety of PCR methods were used to enable detection of DNA from different pathogens (Table 1). PCR products were sequenced (Eurofins Genomics, Ebersberg, Germany) for confirmation and species identification, which was performed by BLAST queries against the NCBI database (http://blast.ncbi.nlm.nih.gov/Blast.cgi). Representative *Babesia* sequences were submitted to the NCBI database (accession numbers, KP688578 and KP688579).

**Table 1:** **Primers used to detect** ***Babesia*** **and other tick-borne pathogens by PCR**

**Pathogen**	**PCR principle**	**Gene target**	**Primer name**	**Primer sequence**	**Reference**
*Babesia* spp.	PCR and sequencing	18S rRNA gene	BJ1	5′-GTCTTGTAATTGGAATGATGG-3′	[21]
			BN2	5′-TAGTTTATGGTTAGGACTACG-3′	
*Bartonella henselae*		Riboflavin synthase gene	BartF	5′-ACGGATATCGGTTGTGTTGAGGA-3′	Present study (modified from [22])
			PBH-R2	5′-AGGTATAAAACGCTTTGGTACTTGTAGG-3′	
*Bartonella quintana*		Riboflavin synthase gene	BartF	5′-ACGGATATCGGTTGTGTTGAGGA-3′	Present study
			PBQR	5′-TTACAATAAAGGGCGTGATGAATTTTGTT-3′	
*Borrelia burgdorferi*	Nested PCR	Flagellin gene	Outer1	5′-AATGAATTGGCAGTTCAATC-3′	[23,24]
			Outer2	5′-GCATTTTCWATTTTAGCAAGTGATG-3′	
			Inner1	5′-ACATATTCAGATGCAGACAGAGGTTCTA-3′	
			Inner2	5′-GAAGGTGCTGTAGCAGGTGCTGGCTGT-3′	
*Francisella tularensis*		peptidyl-prolyl trans isomerase and RNA helicase genes	Ft-M19	5′-CCAGTACAAACTCAATTTGGTTATCATC-3′	[25]
			Ft-M19R	5′-TAGTTTCAGAATTCATTTTTGTCCGTAA-3′	
Rickettsia spp.		16S rRNA gene	16SF2	5′-ACGCTATCGGTATGCTTAACACAT-3′	Present study
			16SR2	5′-CAACTTACTAAACCGCCTACGCACT-3′	
Candidatus *Neoehrlichia mikurensis*		16S rRNA gene	NEO-M140F	5′-ATGGAATAGCTGTTAGAAATGAC-3′	Present study
			NEO-630R	5′-CTATCCTCTCTCGATCTCTAGTTT-3′	

A total of 345 pools (one pool per animal) were included in the present study, comprising a total of 661 ticks. All ticks were *I. ricinus* (females, *N* = 533; males, *N* = 78). In all of the 22 sampling locations except for one, one or more pathogens were detected in one or more of the pools (Additional file 1: Table S1, Additional file 2: Figure S2). Of the 345 examined pools, 118 (34%) were found to be infected with one or more human tick-borne pathogens (Additional file 1: Table S1). *Rickettsia* spp. were detected in 16% of the samples, and *Rickettsia helvetica* accounted for 79% of the rickettsial sequences. Three cases (5%) of *Rickettsia massiliae* were identified, while the remainder of the *Rickettsia* (16%) could not be assigned to species level with confidence either due to low (~97%) sequence identity to other rickettsia or due to ambiguous DNA sequence data.

A total of 15% of the samples were positive for *Borrelia burgdorferi* sensu lato, and five sub-species were identified, including *Borrelia afzelii* (39%), *Borrelia garinii* (27%), *Borrelia valaisiana* (17%), *Borrelia spielmanii* (15%), and *Borrelia burgdorferi* sensu stricto (2%).

Likewise, 8% were positive for *Babesia* spp. (82% *Babesia microti* and 18% *Babesia venatorum* (‘EU1’)), 1% for Candidatus *Neoehrlichia mikurensis*, and 0.6% for *Bartonella* spp. *Francisella tularensis* was not detected in any of the ticks.

The four major regions sampled in the study did not appear to differ in terms of the distribution of pathogens detected in ticks. However, *B. venatorum* was found only in Jutland, while *B. microti* was found in both Funen and Jutland (Table 2). In 11/27 (41%) cases, *Babesia* was seen with at least one co-infecting pathogen (Additional file 1: Table S1), including one case of *B. burgdorferi*.

**Table 2:** **Species of** ***Babesia*** **identified in pools of ticks isolated from dogs in relation to geographical location of host and other pathogens identified in the sample**

**Location**	**Sample nos.** ^**1**^	**Species of** ***Babesia***	**Pathogens other than** ***Babesia***
North Jutland	31A, 31G, 31H, 31 K, 31AA	*B. microti*	*B. afzelii*
Mid-West Jutland	6I, 6 Å, 16R, 26A, 28E, 28R, 29A, 29 F	*B. microti, B. venatorum* (EU1)	*R. helvetica*, *Rickettsia* sp., *B. afzelii*
South Jutland	9C, 10 J, 13A, 13C, 19 T, 22 F, 22O, 22Q, 22 T	*B. microti, B. venatorum* (EU1)	*R. helvetica*, *B. afzelii, B. valaisiana, B. burgdorferi*
South Funen	3G, 3Q, 4E, 4 F	*B. microti*	*R. helvetica, N. mikurensis*
NA^2^	105	*B. microti*	*B. afzelii*

^1^For information regarding individual samples, please refer to Additional file 1: Table S1.

^2^Information on location not available.

Analysis of 661 ticks identified *I. ricinus* as the dominating tick species infecting dogs in Denmark, and approximately one third of the ticks contained parasites and/or bacteria potentially pathogenic to humans. *R. helvetica* was identified as the most common pathogen, confirming previous analyses of questing ticks in Denmark [13-15].

The present data add support to a study recently performed to investigate the presence of tick-borne pathogens identified in *I. ricinus* collected in Denmark, France, and The Netherlands, confirming the presence of *Babesia* in *I. ricinus* in Denmark. Using a microfluidic real-time PCR assay targeting 25 bacteria and 12 parasites, Michelet et al. identified two cases of *B. divergens* and 19 cases of *B. venatorum,* analysing 94 pools of Danish questing *I. ricinus* nymphs sampled in the region of Zeeland, Denmark [9]. However, a few remarkable differences between the two studies could be noted. In the study by Michelet et al. [9] no cases of *B. microti* were detected. In the present study, *B. microti* was i) found in 23/345 samples (7%), ii) more common than *B. venatorum*, and iii) distributed all over the sampling region, which, however, did not include the island of Zeeland or any other area east of Funen. On the other hand, *B. divergens* was found in two cases in the study by Michelet et al. [9], while this species remained unidentified in the present study. Hence, all three main species of *Babesia* of potential clinical relevance to humans have been identified in Denmark, although *B. microti* and *B. divergens* may differ significantly in terms of regional distribution in this country. Of particular interest is also the fact that *B. microti* was identified only in Jutland and Funen and not further east in the country. Notably, *B. microti* detected in the present study appeared 100% genetically identical at SSU rRNA gene level to species detected in New England, where human babesiosis due to *B. microti* is not uncommon [16]. It should also be noted that Michelet et al. [9] used different target genes when screening for *Babesia*: While the SSU rRNA gene was used to detect *B. venatorum*, hsp70 and CCTeta were used to detect *B. divergens* and *B. microti*, respectively. It should be investigated if differences in copy number and level of intra-specific genetic diversity may affect the relative diagnostic performance of each marker. Moreover, the CCTeta assay could be applied to DNAs sequence-positive for *B. microti*, in order to evaluate its applicability as a general screening tool.

While human cases of *B. microti* infections detected in Europe may be imported (e.g. [7]), a couple of autochthonous cases have been reported [17,18]. Interestingly, a *B. microti* infection was found in an immunocompetent male with clinical and laboratory evidence of Lyme disease [18], indicating that co-infections by *Borrelia* and *Babesia* may be overlooked. In Europe, babesiosis occurs not only in splenectomised or immunosuppressed patients, but also in healthy individuals [19].

Rodents appear to constitute the main reservoir for *B. microti* [20]. In the present study, we confirmed the presence of *B. microti* in *I. ricinus*; however, it remains unknown whether *I. ricinus* is capable of transmitting *B. microti* to larger mammals, including humans. Nevertheless, seroprevalence data indicate that *B. microti* infections are not uncommon in Europe [19,20], and that they may have a subclinical course in otherwise healthy individuals.

Future studies should monitor the prevalence, distribution, and host specificity of *Babesia* in ticks from different geographical regions in Denmark and neighbouring countries in order to increase awareness and to enable assessment of the potential public health risk of contracting babesiosis in this region where infections may be emerging. Furthermore, it should be investigated, to which extent patients with Lyme disease are seropositive for *Babesia*. Low diagnostic sensitivity related to traditional diagnosis of acute babesiosis can be overcome by the use of *Babesia*-specific PCR, suitable for direct detection of the pathogen in blood samples from patients with relevant exposure and unexplained fever.

## Conclusion

This study is the first to detect of *B. microti* in Danish ticks. We confirmed the presence of *B. microti* in *I. ricinus* collected at multiple locations in Denmark. Our data evinces endemic occurrence of potentially zoonotic *Babesia* in Denmark and confirms *I. ricinus* as a vector of multiple pathogens of public health concern. Awareness of the potential impact of *Babesia* on public health in Scandinavia could be increased by monitoring the prevalence, distribution, and host specificity of *Babesia* in ticks.

## Additional files

Additional file 1: Table S1. Pathogens detected in the study according to sampling location and co-infecting organisms. Additional 2: Figure S1. Map of Denmark (excluding the island of Bornholm) displaying areas of tick sampling by municipality indicated by the numbers 1—15. For cross-reference, please see the Additional file 1: Table S1.

## References

[1] Zhou X, Xia S, Huang JL, Tambo E, Ge HX, Zhou XN (2014). Human babesiosis, an emerging tick-borne disease in the People’s Republic of China. Parasit Vectors.

[2] Stramer SL, Hollinger FB, Katz LM, Kleinman S, Metzel PS, Gregory KR (2009). Emerging infectious disease agents and their potential threat to transfusion safety. Transfusion.

[3] Hildebrandt A, Gray JS, Hunfeld KP (2013). Human babesiosis in Europe: what clinicians need to know. Infection.

[4] Gigandet L, Stauffer E, Douet V, Rais O, Moret J, Gern L (2011). Prevalence of three zoonotic *Babesia* species in *Ixodes ricinus* (Linné, 1758) nymphs in a suburban forest in Switzerland. Vector Borne Zoonotic Dis.

[5] Bonnet S, Michelet L, Moutailler S, Cheval J, Hébert C, Vayssier-Taussat M (2014). Identification of parasitic communities within European ticks using next-generation sequencing. PLoS Negl Trop Dis.

[6] Chauvin A, Moreau E, Bonnet S, Plantard O, Malandrin L (2009). *Babesia* and its hosts: adaptation to long-lasting interactions as a way to achieve efficient transmission. Vet Res.

[7] Holler JG, Röser D, Nielsen HV, Eickhardt S, Chen M, Lester A (2013). A case of human babesiosis in Denmark. Travel Med Infect Dis.

[8] Diuk-Wasser MA, Liu Y, Steeves TK, Folsom–O’Keefe C, Dardick KR, Lepore T (2014). Monitoring human babesiosis emergence through vector surveillance New England, USA. Emerg Infect Dis.

[9] Michelet L, Delannoy S, Devillers E, Umhang G, Aspan A, Juremalm M (2014). High-throughput screening of tick-borne pathogens in Europe. Front Cell Infect Microbiol.

[10] Dautel H, Dippel C, Oehme R, Hartelt K, Schettler E (2006). Evidence for an increased geographical distribution of *Dermacentor reticulatus* in Germany and detection of *Rickettsia* sp. RpA4. Int J Med Microbiol.

[11] Weidner H, Bestimmungstabellen der Vorratsschädlinge und des Hausungeziefers Mitteleuropas (1993). Spektrum Akademischer Verlag.

[12] Hillyard PD (1996). Ticks of North-West Europe, vol. 52. Montford Bridge, UK: The Linnean Society of London and The Estaurine and Coastal Sciences Association; Field Studies Council Publications.

[13] Kantsø B, Svendsen CB, Jensen PM, Vennestrøm J, Krogfelt KA (2010). Seasonal and habitat variation in the prevalence of *Rickettsia helvetica* in *Ixodes ricinus* ticks from Denmark. Ticks Tick Borne Dis.

[14] Svendsen CB, Krogfelt KA, Jensen PM (2009). Detection of *Rickettsia* spp. in Danish ticks (Acari: *Ixodes ricinus*) using real-time PCR. Scand J Infect Dis.

[15] Skarphédinsson S, Lyholm BF, Ljungberg M, Søgaard P, Kolmos HJ, Nielsen LP (2007). Detection and identification of *Anaplasma phagocytophilum*, *Borrelia burgdorferi*, and *Rickettsia helvetica* in Danish *Ixodes ricinus* ticks. APMIS.

[16] Smith RP, Elias SP, Borelli TJ, Missaghi B, York BJ, Kessler RA (2014). Human babesiosis, Maine, USA, 1995–2011. Emerg Infect Dis.

[17] Hildebrandt A, Franke J, Meier F, Sachse S, Dorn W, Straube E (2010). The potential role of migratory birds in transmission cycles of *Babesia* spp., *Anaplasma phagocytophilum*, and *Rickettsia* spp. Ticks Tick Borne Dis.

[18] Meer-Scherrer L, Adelson M, Mordechai E, Lottaz B, Tilton R (2004). *Babesia microti* infection in Europe. Curr Microbiol.

[19] Martinot M, Zadeh MM, Hansmann Y, Grawey I, Christmann D, Aguillon S (2011). Babesiosis in immunocompetent patients. Europe Emerg Infect Dis.

[20] Yabsley MJ, Shock BC (2013). Natural history of zoonotic *Babesia*: Role of wildlife reservoirs. Int J Parasitol Parasites Wildl.

[21] Casati S, Sager H, Gern L, Piffaretti JC (2006). Presence of potentially pathogenic *Babesia* sp. for human in *Ixodes ricinus* in Switzerland. Ann Agric Environ Med.

[22] Bereswill S, Hinkelmann S, Kist M, Sander A (1999). Molecular analysis of riboflavin synthesis genes in *Bartonella henselae* and use of the ribC gene for differentiation of *Bartonella* species by PCR. J Clin Microbiol.

[23] Clark K, Hendricks A, Burge D (2005). Molecular identification and analysis of *Borrelia burgdorferi* sensu lato in lizards in the southeastern United States. Appl Environ Microbiol.

[24] Johnson BJ, Happ CM, Mayer LW, Piesman J (1992). Detection of *Borrelia burgdorferi* in ticks by species-specific amplification of the flagellin gene. Am J Trop Med Hyg.

[25] Byström M, Böcher S, Magnusson A, Prag J, Johansson A (2005). Tularemia in Denmark: identification of a *Francisella tularensis* subsp. *holarctica* strain by real-time PCR and high-resolution typing by multiple-locus variable-number tandem repeat analysis. J Clin Microbiol.

