# Supplementary material for: Babesia spp. and other pathogens in ticks recovered from domestic dogs in Denmark
Source: Parasit Vectors. 2015 May 8;8:262. doi: 10.1186/s13071-015-0843-0 (PMC4425907; doi:10.1186/s13071-015-0843-0)
Supplement: Additional 2: Figure S1. — Map of Denmark (excluding the island of Bornholm) displaying areas of tick sampling by municipality indicated by the numbers 1—15. For cross-reference, please see the Additional file 1: Table S1. [file 13071_2015_843_MOESM2_ESM.pptx]

## Slide 1
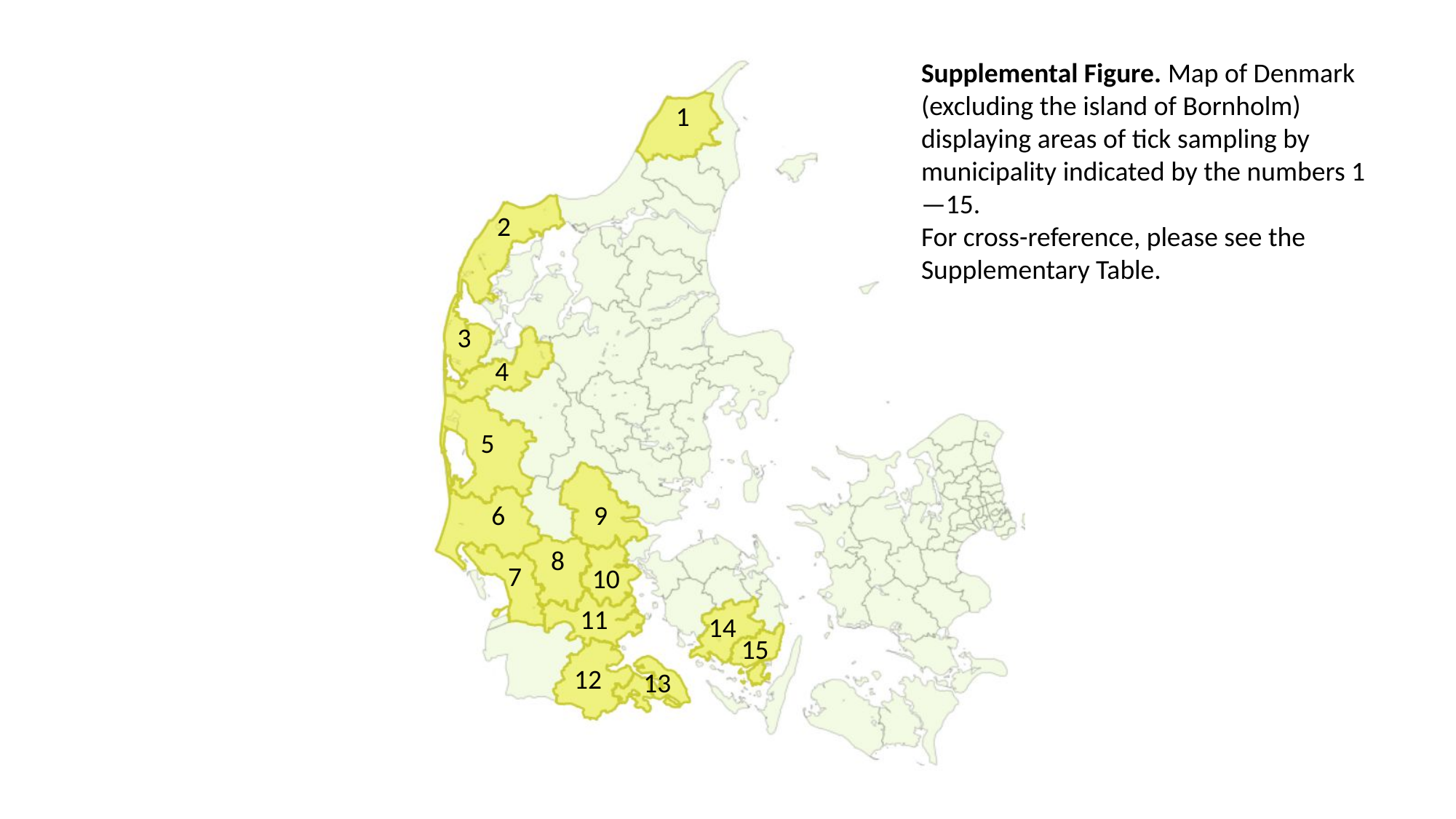

Supplemental Figure. Map of Denmark (excluding the island of Bornholm) displaying areas of tick sampling by municipality indicated by the numbers 1—15.
For cross-reference, please see the Supplementary Table.
1
2
3
4
5
6
9
8
7
10
11
14
15
12
13
